# Undeveloped Female Sexual Organs, Coitus per Urethram

**Published:** 1914-06

**Authors:** 


**Undeveloped Female Sexual Organs, Coitus per Urethram.** C. H. Johnson and Thomas S. Blair reports a case each, June and July issues, respectively, 1913, Medical World.

